# Exploration of biophoton characteristics of fresh *Isatis indigotica* fort leaves under salt and drought stresses and the feasibility analysis for the quality prediction of *Isatidis Folium*

**DOI:** 10.3389/fpls.2025.1523636

**Published:** 2025-03-12

**Authors:** Zhiying Wang, Baorui Cao, Yueyue Ma, Weifan Xu, Jialei Fu, Zhongwen Zhang, Jinxin Du, Tingting Deng, Jingxiang Pang, Meina Yang, Jinxiang Han

**Affiliations:** ^1^ Department of Clinical Pharmacy, The First Affiliated Hospital of Shandong First Medical University & Shandong Provincial Qianfoshan Hospital, Shandong Medicine and Health Key Laboratory of Clinical Pharmacy, Jinan, China; ^2^ Biomedical Sciences College & Shandong Medical Biotechnology Research Center, National Health Commission Key Laboratory of Biotechnology Drugs, Shandong First Medical University & Shandong Academy of Medical Sciences, Jinan, China; ^3^ College of Traditional Chinese Medicine, Shandong University of Traditional Chinese Medicine, Jinan, China; ^4^ Institute of Traditional Chinese Medicine Pharmacology, Shandong Academy of Traditional Chinese Medicine, Jinan, China; ^5^ Department of Endocrinology and Geriatrics, The Third Affiliated Hospital of Shandong First Medical University, Jinan, China

**Keywords:** *Isatis indigotica* Fort, *Isatidis Folium*, biophoton emission, stress condition, quality evaluation, active ingredient content, antibacterial activity

## Abstract

**Introduction:**

*Isatidis Folium*, derived from the dried leaves of *Isatis indigotica* Fort, has been used for centuries as a traditional Chinese herb with antibacterial and antiviral properties. However, both the cultivation conditions and the growth status of *Isatis indigotica* Fort have been negatively affected by climatic and environmental degradation, which has made it challenging to accurately assess the quality of *Isatidis Folium*. The current quality control system for *Isatidis Folium* lacks precision and comprehensive identification indices, and importantly, the cultivation process has not been integrated into this system.

**Methods:**

In this study, we proposed a novel method to distinguish between different stress subtypes in *Isatis indigotica* Fort based on biophoton emission and attempted to explore the potential relationship between the biophoton characteristics of fresh *Isatis indigotica* Fort leaves and the quality of *Isatidis Folium*. The delayed luminescence (DL) and spontaneous photon emission (SPE) characteristics of fresh *Isatis indigotica* Fort leaves under different stress conditions were detected using a biophoton detection system. An attempt was made to differentiate samples subjected to various stress treatments using biophoton characteristic parameters. Additionally, the content of active ingredients was determined by ultra-high performance liquid chromatography, and the inhibitory activity against Escherichia coli and Staphylococcus aureus was evaluated to identify the quality of *Isatidis Folium*. Several physiological indicators of fresh *Isatis indigotica* Fort leaves, including the photosynthetic pigment content, relative electrical conductivity, and reactive oxygen species production rate were also determined.

**Result:**

The differences in physiological indices, active ingredient content, and inhibitory activity indicated that the stress conditions significantly inhibited the growth status of *Isatis indigotica* Fort leaves and the herbal quality. Meanwhile, biophoton characteristic parameters were obtained that could accurately and efficiently distinguish fresh *Isatis indigotica* Fort leaves between different stress subtypes: initial intensity of DL and counts per second of SPE. Both characteristic parameters were highly correlated with the physiological indicators and quality of *Isatidis Folium*.

**Discussion:**

This study has preliminarily demonstrated the feasibility of utilizing biophoton detection technology for the quality evaluation of *Isatidis Folium* during cultivation for the first time and provided an improved method for distinguishing samples of various qualities.

## 1 Introduction

The traditional Chinese herb, *Isatidis Folium*, belongs to the cruciferous plant *Isatis indigotica* Fort [Brassicaceae], specifically the leaf part of the entire plant, which has been extensively utilized in Eastern countries for centuries. *Isatis indigotica* Fort has been officially recognized as the only source of *Isatidis Folium* since the 1985 edition of the Chinese Pharmacopoeia. *Isatis indigotica* Fort primarily produced in the Yellow River basin north of the Yangtze River (Chen et al., 2022). It is cold-resistant, warmth-favoring, and relatively drought-tolerant, but does not tolerate waterlogging (Commission, C. P, 2020). *Isatidis Folium* has the effects of clearing heat, detoxicating, cooling blood, and eliminating spots. It has been proven to have various pharmacological activities including antibacterial, anti-inflammatory, anti-endotoxin, and anticancer effects (Chung et al., 2024), and is frequently used to alleviate the symptoms of colds, fevers, sore throats, viral hepatitis, and epidemic encephalitis B in clinical (Wu et al., 2018; Chen et al., 2022). Previous studies have shown that *Isatidis Folium* primarily contains active ingredients such as alkaloids (Liu et al., 2018; Ji et al., 2013; Xiong et al., 2022; Yuan et al., 2023), flavonoids (Jiang et al., 2015; Yu et al., 2018), organic acids (Mao, 2018; Yan et al., 2024), and polysaccharides (Guo et al., 2016). Among these, alkaloids, especially indole alkaloids, have been extensively studied and have been found to have significant biological activities, and are often used as quality control indicators for *Isatidis Folium*. Indole alkaloids mainly include indigo, indirubin, and tryptanthrin (Xu et al., 2021). Indirubin is the main active ingredient of *Isatidis Folium* (Chen et al., 2022), which has been demonstrated to have excellent antibacterial, anti-inflammatory, anti-leukemic, and immune-enhancing activities (Wu et al., 2016; Chuang et al., 2024). Indigo has been proven to be liver-protective, anti-inflammatory, and antimicrobial (Zhang et al., 2020). Tryptanthrin is an alkaloid with an indole-quinazoline structure that has pharmacological activities including antibacterial, antiviral, anti-inflammatory, and anticancer (Alam et al., 2025).

The quality control of *Isatidis Folium* is challenging due to the influence of several factors, including its origin, germplasm, the growing environment, and the harvest season. Environmental factors are the core external factors affecting the formation of herbal quality and are crucial for the efficacy of Chinese herbs. *Isatis tinctoria* Fort is susceptible to external conditions such as soil, moisture, salinity, etc., which can affect the quality of *Isatidis Folium*. Due to the current global warming, soil desertification, and increased salinization, salt stress and drought are the two major abiotic stresses on herbal plants, significantly limiting the quality of Chinese herbs. Additionally, the use of artificial organic pesticides and fertilizers has increased the levels of organic phosphorus, organic nitrogen, heavy metal ions, microorganisms, and other residues in *Isatidis Folium*, which can result in a reduction in herbal quality (Gao and Dong, 2010). Fourteen batches of *Isatidis Folium* and corresponding tablets from different origins and environments were investigated and the results showed that *Isatidis Folium* samples had high ash content and significantly insufficient active ingredient content (La and Hu, 2009). This indicates that the quality of *Isatidis Folium* products in the market is inconsistent and below the required standard. As a result, traditional Chinese herbal preparations based on *Isatidis Folium*, such as Qinghuo tablets, Xiaowen Jiedu oral liquid, and Fufangdaqingye mixture, failed to meet the required quality standards. Therefore, the clinical efficacy would be negatively affected.

According to the Chinese Pharmacopoeia 2020, the indirubin content of *Isatidis Folium* should be determined using high-performance liquid chromatography (HPLC) as the quality evaluation method (Commission, C. P, 2020). Currently, reverse-phase high-performance liquid chromatography (RP-HPLC) is the primary method used to quantify the primary active ingredients in *Isatidis Folium* (Xiao et al., 2015). In related literature, the Box-Behnken response surface method was used to optimize the indices and strategies for the determination of the quality standard content of *Isatidis Folium* in the Chinese Pharmacopoeia, and the contents of indigo and indirubin were simultaneously quantified by HPLC, which provided a new method for the quality control of *Isatidis Folium* and tablets (Wang et al., 2024). The contents of 4-hydroxyquinazoline, 2,4-hydroxyquinazoline, syringic acid, tryptanthrin, indigo, and indirubin of *Isatidis Folium* were quantified based on the multi-component content determination, and the quality evaluation method based on HPLC fingerprinting was then established (Li et al., 2019). Due to the extreme complexity of the chemical composition of Chinese herbs, it is tough to base quality control on a clear study of all the components. The current quality measurement therefore typically relies more on some ingredients with significant pharmacological activity (Sun et al., 2021), which is still the most common and universal consensus in herbal quality control. Pharmacological activity testing can directly reflect the potential efficacy of certain aspects of Chinese herbs and is the more widely used and recognized quality identification method. However, it is more cumbersome and time-consuming than quantification of medicinal composition, and is also difficult to explore all the efficacy completely. Therefore, it is crucial to reinforce the quality control of *Isatidis Folium* and establish a simpler quality evaluation method that agrees with the holistic view of traditional Chinese medicine and is tailored to the specific attributes of *Isatidis Folium*. This will help enhance the quality evaluation system for this Chinese herb.

Biophoton emission is a universal phenomenon in living organisms (Zhou, 2019). It is believed to be generated during the transition from high-energy to low-energy states of biological molecules and conveys intricate information about the internal dynamics of the living system (Popp et al., 1988; Gu, 2012). It changes with the physiological state of the organism and is highly sensitive to various physiological and biochemical conditions within the organism and the external environment. Biophoton emission can closely reflect cell division, cell death, information transfer, functional regulation, photosynthesis, and other physiological processes (Wang et al., 2015). Two different types of biophoton emission have been identified. The first is the autonomous luminescence of organisms, known as spontaneous photon emission (SPE). Its luminosity is remarkably low, with a photon emission rate of only hundreds to thousands per unit time and area, depending on the intrinsic properties of the sample tested. The other is delayed luminescence (DL), which occurs when a sample in a steady state is suddenly exposed to a stimulus such as a light source, its luminescence intensity suddenly increases, followed by a trend similar to hyperbolic decay until the luminescence intensity returns to the initial steady state. Biological samples from different sources exhibit different SPE and DL characteristics due to their inherent properties. Therefore, the biophoton characteristics of the biological samples can be used to determine the exact state and comprehensive internal information of the samples. Previous studies (Sun et al., 2021) have revealed that DL is related to the contents of anthraquinone derivatives, suggesting that the DL might be able to reflect the impact of environmental factors on the quality of *Rhei Radix Et Rhizoma* and be used to evaluate the quality of Chinese herbs. Before this, relevant laboratory studies were conducted on screening different varieties of fresh Chinese herbs at different growth ages using SPE analysis (Zhao et al., 2017). The research results showed that SPE can be used to characterize the active substance content in Chinese herbs of different growth ages and varieties. Therefore, this evaluation could contribute to achieving quality control for Chinese herbs in the future.

In this study, we established two types of stress models: salt stress and drought stress. The biophoton characteristics of fresh *Isatis indigotica* Fort leaves were subjected to systematic analysis to ascertain their behavior under different stress conditions. By combining the biophoton characteristic parameters with physiological indicators, the content of active ingredients, and minimum inhibitory concentration (MIC), we investigated the underlying reasons for the differences in the biophoton characteristics of *Isatis indigotica* Fort leaves and its intrinsic relationship with the quality of *Isatidis Folium* under varying stress conditions. We obtained the biophoton characteristic index parameters that can characterize the different attributes of *Isatis indigotica* Fort leaves and clarified the feasibility of the biophoton technology for the quality prediction of *Isatidis Folium*.

## 2 Materials and methods

### 2.1 Herbal cultivation and stress treatments

*Isatis indigotica* Fort was planted in planting boxes (0.4 m × 1.2 m × 0.5 m) in the herbal garden of Shandong First Medical University (116° 8’ 6” N, 116° 8’ 6” E, Jinan, Shandong Province, China) on August 20, 2022. Before sowing, the soil was turned to a depth of 30 cm, and sheep manure was applied as a base fertilizer. Then, 3-4 cm deep trenches were dug for sowing, with a spacing of 5-8 cm between plants and 15 cm between rows. A transparent thermal insulation shed was constructed outside of the boxes. No shading treatment was applied throughout the planting process and 24-hour ventilation was maintained. Drought stress and salt stress were applied to *Isatis indigotica* Fort and each stress condition followed the principle of a single control. Seeds were purchased from the Bozhou Changnong Chinese Medicinal Materials Planting Co., Ltd, and *Isatis indigotica* Fort was identified by researcher Fu Jialei, confirming that they met the identification standards as specified in the 2020 edition of the Chinese Pharmacopoeia (Commission, C. P, 2020). The technical specifications for planting, collection, storage, and primary processing were conducted by the Law of the People’s Republic of China on Traditional Chinese Medicine and other relevant regulations (Committee, N. S, 2017).

#### 2.1.1 Salt stress treatments

After sowing, *Isatis indigotica* Fort was grown under the same conditions. Twenty days after the seedling emergence, four boxes of uniformly grown and healthy *Isatis indigotica* Fort were selected for subsequent cultivation. Four different salinity gradients of 0 mmol/L (the control group), 60 mmol/L (low salinity), 120 mmol/L, and 180 mmol/L (high salinity) were established for the salt stress treatment. Each box of *Isatis indigotica* Fort was fixed at approximately 50 plants after seedling and interplanting. To avoid excessive osmotic stress, the salinity was gradually increased, starting at 1/3 of the predetermined concentration for the first stress, 2/3 for the second stress, and reaching the full predetermined concentration for the third stress. Salt stress was applied once a week for one month, with each injection of twice the amount of water held by the soil (Mi et al., 2018). To maintain soil salinity at the respective gradient levels throughout the stress period, soil samples were collected daily from each treatment group and air-dried. Soil salinity was then determined using the conductivity method (Song, 2011).

#### 2.1.2 Drought stress treatments

This experimental study was carried out from 1 September to 1 November. After seed emergence, two boxes of *Isatis indigotica* Fort with consistent growth status were selected for subsequent cultivation. Each box contained approximately 50 plants. Two irrigation treatments were established: the control group (adequate water supply) with a soil water content of 70-90% and drought stress with a soil relative water content of 30-50%. The control group was irrigated three to four times a week with 3 L of well water and the drought stress group was irrigated two to three times a week with 1 L. To maintain the soil water content within the set range, daily evaporation was compensated for by measuring soil moisture each evening through the gravimetric method (Ma, 2022) and adjusting water application accordingly. This approach ensured that all treatments adhered to their designated moisture conditions. The drought stress treatment lasted for two months.

### 2.2 Biophoton detection

#### 2.2.1 Sample preparation

*Isatis indigotica* Fort plants grown under different stress conditions were utilized as the experimental samples, collected from the boxes in the herbal garden. Fresh samples were collected in the morning and afternoon to avoid exposure to the midday sun and to prevent the sampling and experiments during extreme weather conditions. This was done to ensure the natural environment was as consistent as possible when collecting experimental samples. Samples were randomly collected and immediately stored in ice boxes after collection. The fresh samples were cleaned with double distilled water, blotted dry on blotting paper, and photographed for recording. Subsequently, the samples were placed in the darkroom of the instrument for one hour for dark adaptation. Each experiment maintained a consistent time interval between sample collection and dark adaptation.

#### 2.2.2 Measurement system and biophoton detection

**Figure 1:**
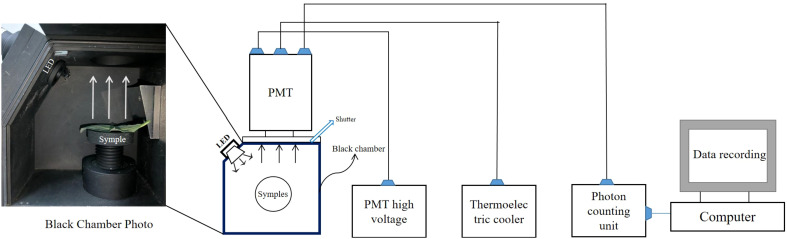
The schematic diagram of the biophoton detection system used in this study.

Before the measurements, a constant room temperature of 20°C was established and the YPMS-2 biophoton detector (Miluna, the Netherlands) was switched on for a two-hour cooling period. This system consists of a black chamber, a photomultiplier tube (PMT), a photomultiplier tube high-voltage power supply type PMS20S (Sens-Tech), a photon counting unit C9744 (Hamamatsu Photonics K.K., Iwata, Japan), a thermoelectric cooler and a computer equipped with photon counting data software (**Figure 1**). The core component of this detection system is the PMT with a shutter in front of it to avoid any interference from external light. After dark adaptation, the SPE characteristics of the sample were examined. The measurement time for each detection point was set at one second, with a continuous measurement period of ten minutes and a total of 600 detection points. The DL characteristics and the excitation were conducted using a light-emitting diode (LED) (7 mm, 10 W, type LZ4-00MD00; LED Engine Inc., United States). The samples were illuminated by the LED for 15 seconds and then detected continuously for 5 minutes. Three replicate DL experiments were performed for each sample. After the DL detection, the background noise was detected for 10 min, the sample weight was weighed and the thickness was also measured. To minimize contingency effects, six independent experimental replications were conducted for each group, with all six replicates completed within a single day.

#### 2.2.3 Data processing

To find the parameters representing the biophoton characteristics and then to quantitatively compare the differences under various stress conditions, we characterized the SPE properties with counts per second (CPS). In addition, to calculate the DL characteristics of the samples, all the photon counts measured during the 300 s of each decay curve were used according to the Gu parameter function (Equation 1). Six parameters were obtained from the Gu parameter equation. They are the three “Gu parameters” (A, B, C), the initial intensity (I_0_) of DL, the average intensity of the entire DL process (I_W_), and the decay time (T). The values of A, B, and C are derived while fitting the DL kinetic curves using the Gu parameter equation. While I_0_, I_W_, and T are macroscopic quantities calculated based on A, B, and C. These parameters can well express the characteristics of the DL decay curve. Subsequently, we used the least absolute shrinkage and selection operator (LASSO) to screen pivotal parameters that could be utilized to characterize the biophoton characteristics of fresh *Isatis indigotica* Fort leaves. The Gu parameter equation (Equation 1) and the equations for each parameter (Equations 2–5) are provided blow (Gu, 2012; Sun et al., 2017):


(1) $$I(t)=\text{A}\;\times \;\csc h^{2}(\frac{t}{B}+\text{C})$$

(2) CPS=N–n

(3) $$T=B\times \{ln(\sqrt{m}\times sinhC+\sqrt{\left(m\times sinh^{2}C+1\right)})-C\}$$

(4) $$I_{0}=\text{A}\;\times \csc h^{2}\text{C}$$

(5) $$I_{w}=\frac{A\times B}{W}\times [\coth C-\coth(\frac{W}{B}+C)]$$

In Equation 1, t is the time; A is an intensity parameter dependent on the sample’s nature, system structure, and illumination conditions; B is a characteristic time, related only to the nature of the sample itself; and C is a phase factor determining the initial state of the sample, which is sensitive to the I_0_ of the DL. In Equation 2, N is the average count rate of the sample, and n is the background count rate of the instrument without a sample. The light intensity decays to I_t_ = I_0_/m when the time is T. The value of the factor “m” ranges from 2 to 4 and in this study m = 3. In Equation 5, W is the total measurement time. The strength indicators I_0_, I_W_, and CPS were homogenized by multiplying the thickness and dividing by the weight of the corresponding sample.

### 2.3 Multi-component content determination based on ultra-high performance liquid chromatography

#### 2.3.1 Sample preparation for content determination

After the fresh leaves were collected, they were washed with double distilled water, blotted with absorbent papers, and dried in a drying box (HAIYU) at 60°C for 24 hours. The dried leaves were then ground using an 800C multifunction grinder (Dongguan Fanta Electrical Co., Ltd.) and sieved through a 65-mesh Chinese medicine sieve (0.212 mm aperture). Finally, they were placed in centrifuge tubes and stored in a cool and dry place.

A total of 3 g of *Isatidis Folium* powder was accurately weighed and transferred to a beaker. 50 mL of chloroform was added and the mixture was thoroughly shaken and weighed. The sample was then heated and refluxed for 30 minutes, cooled, and reweighed and chloroform was added to make up for the loss in weight. The mixed solution was then thoroughly shaken, filtered, and concentrated to dryness. An appropriate amount of methanol-chloroform mixture (8:2, v/v) was added to dissolve the residue. The solution was then transferred to a 10 mL volumetric flask, diluted to the mark with the same solvent, and thoroughly mixed to obtain the test solution of *Isatidis Folium*.

#### 2.3.2 UPLC analysis

The UPLC analysis was performed using a Waters Acquity UPLC (Waters, Milford, MA, USA) that was equipped with an ACQUITY UPLC sample organizer, a chromatographic column manager, a column temperature box with heating and cooling functions, a binary solvent manager and sample manager, a photodiode array, and an evaporative light scattering detector. The chromatographic analysis was performed using an ACQUITY UPLC BEH C_18_ column (150 mm × 2.1 mm, 1.7 μm) with a mobile phase of 0.1% phosphoric acid solution (A) - methanol (B). A gradient elution was used as follows: 0 min-3.5 min, 75% A-74% A; 3.5 min-5 min, 74% A-65% A; 5 min-6 min, 65% A-65% A; 6 min-10 min, 65% A-55% A; 10 min-18.5 min, 55% A-30% A; 18.5 min-25 min, 30% A-12% A; 25 min-26 min, 12% A-12% A; 26 min-26.01 min, 12% A-75% A; 26.01 min-33 min, 75% A-75% A. The detection time was 35 min. The injection volume was 10 μL and the flow rate was 0.3 mL/min. The column temperature was maintained at 35°C and the sample cell temperature was set at 25°C.

### 2.4 Detection of MIC

#### 2.4.1 Activation of bacterial strains

*Escherichia coli* (*E. coli*) and *Staphylococcus aureus* (*S. aureus*) were removed from the -80℃ refrigerator and streaked on solid LA culture media on an ultra-clean workbench. The bacterial strains with similar morphology to be determined were picked up with an inoculation loop and inoculated onto a liquid LB media. Bacterial strains were incubated at 37°C for 2-6 h to OD_600_ = 0.7, containing approximately 1-2×10^8^ CFU/mL (calibrated with sterile 0.85% saline). The bacterial solution was diluted 100-fold to obtain a solution containing approximately 1-2 × 10^6^ bacteria.

#### 2.4.2 Preparation of *Isatidis Folium* Decoction

A sample of 40.96 g of *Isatidis Folium* and 200 mL of double-distilled water were placed in a casserole, soaked together for 30 min, and decocted on a 2200 W induction cooker. After cooling, the power was reduced to 800 W, and the total decoction time was 15 min. At the end of the decoction, the decoction was filtered through a 6-layer gauze. The filter residue was placed in a casserole, 200 mL of double-distilled water was added, and the above process of decoction and filtration was repeated. The two filter liquors were combined and placed in a casserole, and the power of the induction cooker was set at 2200 W for decoction. After boiling, the power was reduced to 800 W. Then the heating process was continued until the decoction volume was concentrated to 10 mL, i.e., the final concentration was 4.96 g/mL. The decoction was filtered using a 0.22 μm sterile filter membrane in an ultra-clean bench to remove bacteria and large particles and then stored in a refrigerator at 4°C.

#### 2.4.3 Detection of MIC values

100 μL of LB medium was added to each of 8 adjacent wells of the 96-well plate respectively. 100 μL of the drug solution was added to the first well and mixed thoroughly, then 100 μL of the mixture was taken and added to the second well. The mixture of the second well was mixed thoroughly and then 100 μL of the mixture was taken and added to the third well, and so on. The mixture of the eighth well was mixed thoroughly and 100 μL of the mixture was taken and discarded. Then, 100 μL of bacterial solution was added to each of the eight wells respectively, thus creating samples to be tested with different drug concentration gradients: 1024, 512, 256, 128, 64, 32, 16, 8 μg/mL. 100 μL of LB medium and 100 μL of bacterial solution were added to the ninth well as a positive control group. 100 μL of LB medium and 100 μL of drug solution were added to the tenth well as a negative control. Four samples were prepared in parallel for four replicates. The 96-well plate was incubated in a shaker incubator at 37 ℃ for 18 h. The absorbance at 600 nm of each well was detected with an enzyme marker, and 80% of the bacterial concentration of the positive control group was used as the endpoint for determining the MIC value, and the concentration of the drug solution which was closest to this endpoint was the MIC value of *Isatidis Folium*.

### 2.5 Determination of the photosynthetic pigment content

Fresh samples were collected, washed with double distilled water, and dried with absorbent papers. The leaves were cut into pieces, avoiding the main veins, and 0.1000 g of the crushed leaves were accurately weighed and transferred to a 50 mL centrifuge tube. 15 mL of extract (a 1:1 volume mixture of ethanol and acetone) was added to the centrifuge tube. The mixture was then placed in complete darkness for 24 hours until the leaves had completely faded. During this period, the mixture was shaken two to three times. The absorbance of the extract at 474, 642, and 665 nm was later determined using a U-3900H UV/VIS spectrophotometer (Hitachi High-Tech Science Corporation, Tokyo, Japan). Three biological replicates were made for each treatment. The contents of chlorophylls and carotenoids were determined according to the method of Huang and were calculated using Equations 6–9 (Huang et al., 2013):


(6) $${\text{C}}_{\text{a}}=9.99\times {\text{A}}_{665}-0.0872\times {\text{A}}_{642}$$

(7) $${\text{C}}_{\text{b}}=17.7\times {\text{A}}_{642}-3.04\times {\text{A}}_{665}\;$$

(8) $${\text{C}}_{\text{c}}=4.92\times {\text{A}}_{474}-0.0255\times {\text{C}}_{\text{a}}-0.225\times {\text{C}}_{\text{b}}$$

(9) C=C'×Vm

C_a_, C_b_, and C_c_ are the concentrations (mg/L) of chlorophyll a, chlorophyll b, and carotenoids, respectively. A_s_ represents the absorbance of the extraction solution at the corresponding wavelength. In Equation 9, C is the content (mg/g) of the photosynthetic pigment, C’ is the concentration of the corresponding photosynthetic pigment, V is the volume (L) of the extraction solution, and m is the weight (g) of the leaf samples.

### 2.6 Determination of the relative electrical conductivity

The freshly collected samples were washed with double distilled water and subsequently dried with absorbent papers. Ten leaf discs were quickly punched from different parts of the same leaf using a 6 mm punch, avoiding the main veins. These leaf discs were then placed in a conical flask containing 30 mL of ultrapure water (Millipore, Boston, MA, USA). This mixture was shaken on a shaker at 150 rpm for 12 h and the conductivity was then measured using a DDS-11A conductivity meter. After boiling in a water bath for 30 min and cooling to room temperature, the mixture was shaken to re-determine the conductivity. Three replicate experiments were performed for each treatment. The REC was calculated using Equation 10:


(10) $$E=\frac{E_{2}-E_{1}}{E_{3}-E_{1}}$$

E_1_, E_2_, and E_3_ represent the conductivity of double distilled water, samples, and samples after boiling water bath, respectively.

### 2.7 Determination of mitochondrial reactive oxygen species generation rate

0.1 g of fresh samples were weighed and placed in a mortar, to which 1 mL of Tris-HCl buffer 1 (containing sucrose and EDTA) and 10 μL of PMSF solution were added. The leaf pieces were ground and homogenized, and the mixture was centrifuged at 600 rpm for 5 min at 4°C. The supernatant was transferred to a new centrifuge tube and centrifuged at 11,000 rpm for 10 min at 4°C. The precipitate was resuspended in 200 μL of Tris-HCl buffer 2 (containing potassium chloride, magnesium chloride hexahydrate, and glucose) and gently mixed with a pipette gun. 50 μL of malic acid, 50 μL of pyruvic acid, 50 μL of succinic acid, and 30 of μL DCFH-DA were added to the determination tube and the blank tube, respectively. In addition, 20 μL of mitochondrial suspension was added to the determination tube and 20 μL of Tris-HCl buffer 2 was added to the blank tube. They were mixed well and incubated at 37°C for 15 min in the dark. The Tristar²S LB 942 multifunctional enzyme labeler (Berthold, Bad Wildbad, Germany) was preheated for 30 min, and the excitation and emission wavelengths were set to 499 nm and 521 nm, respectively. Fluorescence intensity was measured at 37°C within 10 min. Three replicate experiments were performed for each treatment. The change in fluorescence intensity over time was analyzed by fitting a linear regression to calculate the slope (k). The actual rate of mitochondrial ROS production (u/s/g) was then determined using Equation 11:


(11) $$v=\frac{10\times (k_{1}-k_{2})}{g}$$

v represents the production rate of ROS; k_1_ and k_2_ represent the slope of the linear regression line for the data points pertaining to the fluorescence intensity of the sample in the detection tube and the blank tube over time, respectively; g represents the weight of the samples.

### 2.8 Data Analysis

Principal component analysis (PCA) was used to indicate the degree of discrimination between the biophoton characteristics of the control group and stress groups. The mean ± standard deviation (SD) values of the samples were statistically analyzed using GraphPad Prism 9.5.0 for Windows (GraphPad Software, La Jolla, CA, USA, www.graphpad.com). Data from samples under different stress conditions were compared using a two-tailed unpaired Student’s t-test. Spearman correlation analysis was performed to explore the potential relationships between biophoton signature parameters and active ingredient content, MIC values, and physiological indices. In all statistical tests, *P* ≤ 0.05 was considered statistically significant.

## 3 Results

### 3.1 Growth status of *Isatis indigotica* Fort leaves under different stress conditions

**Figure 2:**
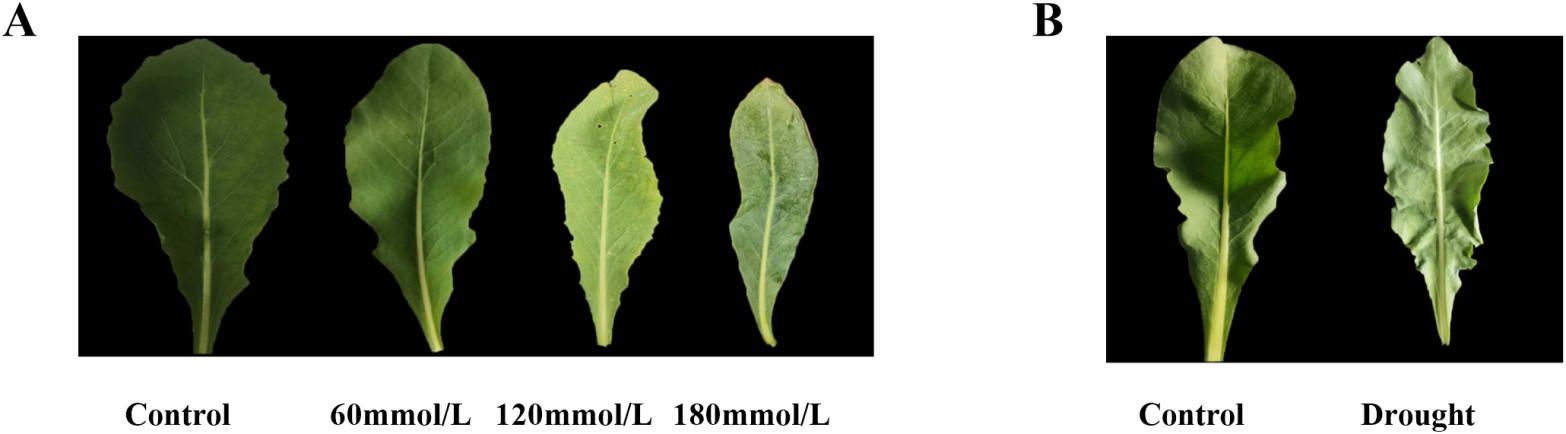
The fresh *Isatis indigotica* Fort leaves under different stress conditions. **(A)** Salt stress. **(B)** Drought stress.

**Figure 2** shows the fresh *Isatis indigotica* Fort leaves under different stress conditions. Under these stressors, the growth of leaves exhibited varying degrees of inhibition. In the salt stress groups, the increasing salinity gradually inhibited the leaf development, resulting in gradual chlorosis, marginal curling, withering, and even the emergence of sporadic necrotic spots. In the drought stress group, the leaves were small and thin, excessively soft, and wrinkled.

### 3.2 Biophoton characteristics of fresh *Isatis indigotica* Fort leaves

#### 3.2.1 Raw biophoton data

**Figure 3:**
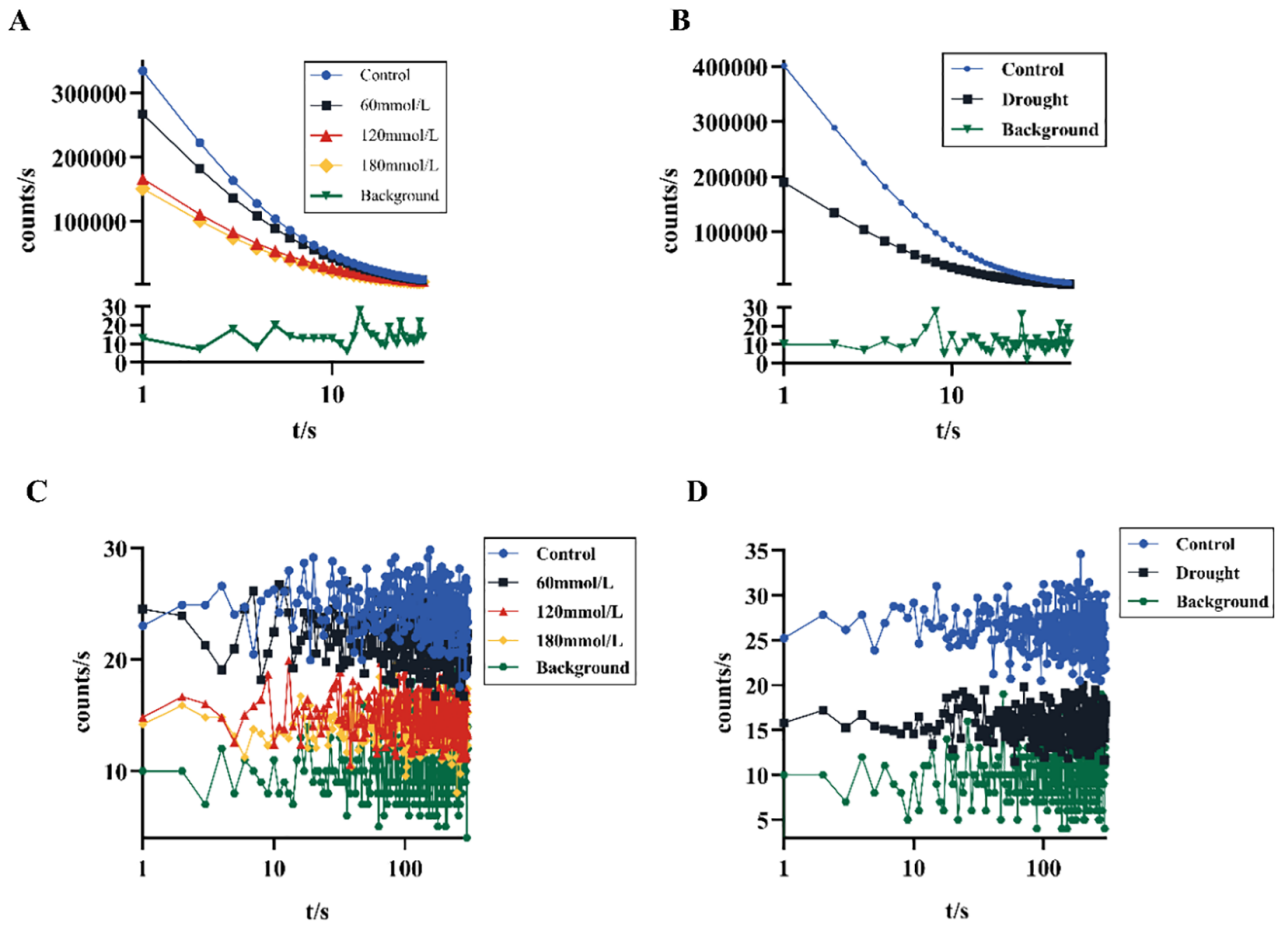
Biophoton characteristics of fresh *Isatis indigotica* Fort leaves under different stress conditions. **(A, B)** DL kinetic curves of samples under salt and drought stress treatment groups, respectively. **(C, D)** SPE characteristics of samples under salt and drought stress treatment groups, respectively.

**Figure 3** shows different DL kinetic curves and SPE characteristics of *Isatis indigotica* Fort leaves samples under different stress conditions. Both salt and drought stresses significantly altered the SPE characteristics, which was mainly reflected in the biophoton intensity at the steady state. The biophoton intensity of samples was significantly lower in the high salinity and drought stress groups compared to the control group. In addition, the T and I_0_ of the DL characteristics varied with different samples, and the intensity of DL after stimulation of biological systems tended to be much higher than that of SPE at the steady state. The DL characteristics of samples exposed to both salt and drought stress treatments also significantly changed, especially the parameter I_0_, which shows the most obvious changes. The initial photon emission intensity of the samples under the stress treatments was approximately half that of the control group.

#### 3.2.2 Screening of biophoton parameters

**Figure 4:**
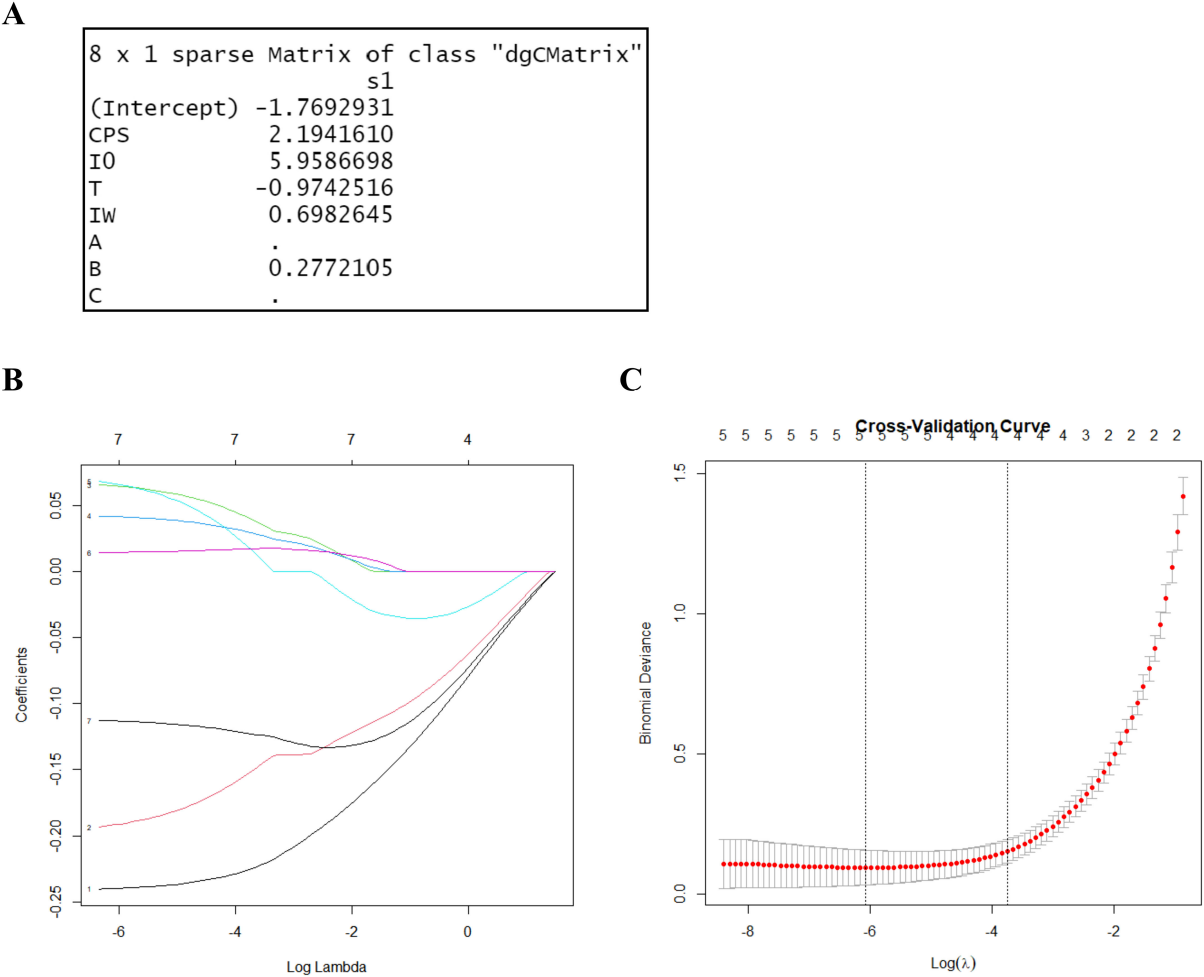
LASSO regression for variable selection. **(A)** LASSO output characteristic coefficients. **(B)** LASSO coefficient path plot. It reflects the relationships between the coefficients of the variables and the value of log lambda. Each curve represents the trajectory of the coefficients of a feature as a function of λ. The horizontal axis represents the value of λ on a logarithmic scale, the vertical axis represents the coefficient of the feature, and different colors represent different features. **(C)** Cross-validation results of the LASSO regression. By verifying the optimal parameter (λ) in the LASSO model, the partial likelihood binomial deviation was plotted against log (λ). The dashed line was determined using the minimum criterion and the one standard error of the minimum criteria (the 1-SE criteria) in selecting the optimal log (λ) values of the features. The best lambda value from cross-validation is 0.002297955.

To differentiate between the control and stress groups, LASSO regression was used to screen for characteristic parameters. In LASSO regression, the range of values of the penalty coefficient is usually gradually reduced from a series of larger values, and the smaller the cross-validated mean square error, the better the model fits. As the penalty coefficients change, the more important the variable, the later the coefficients are compressed to zero. Seven relevant characteristic variables were considered in this study. Based on the training cohort data, three potential indicator parameters were identified: CPS, I_0_, and T. These parameters exhibited relatively high importance coefficients in the LASSO regression model (**Figure 4**).

#### 3.2.3 Analysis of biophoton parameters under different stress conditions

**Figure 5:**
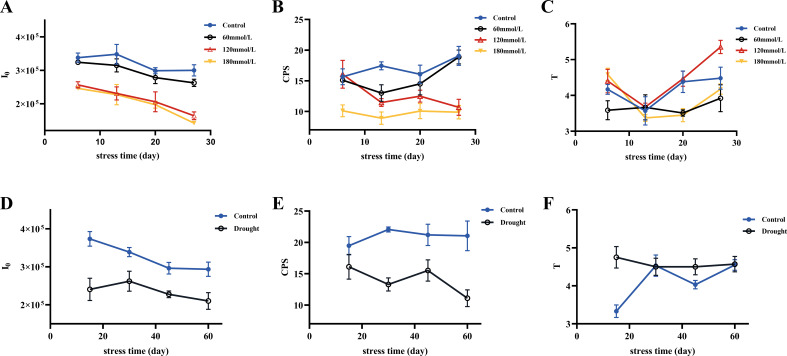
Three selected biophoton characteristic parameters (I_0_, CPS, and T) of fresh *Isatis indigotica* Fort leaves under different stress conditions with stress time. **(A–C)** The changing trends of I_0_, CPS, and T in the salt stress group with stress time, respectively. **(D–F)** The changing trends of I_0_, CPS, and T in the drought stress group with stress time, respectively.

**Figure 5** shows the three biophoton characteristic parameters of *Isatis indigotica* Fort leaves under different stress conditions. High salinity and drought stress conditions induced significant changes in the biophoton parameters. Compared to the control group, both I_0_ and CPS showed a highly significant decrease under these stress conditions, while the low salinity stress group had a relatively minimal effect. The variation trend of T under different stress conditions was not significant.

**Figure 6:**
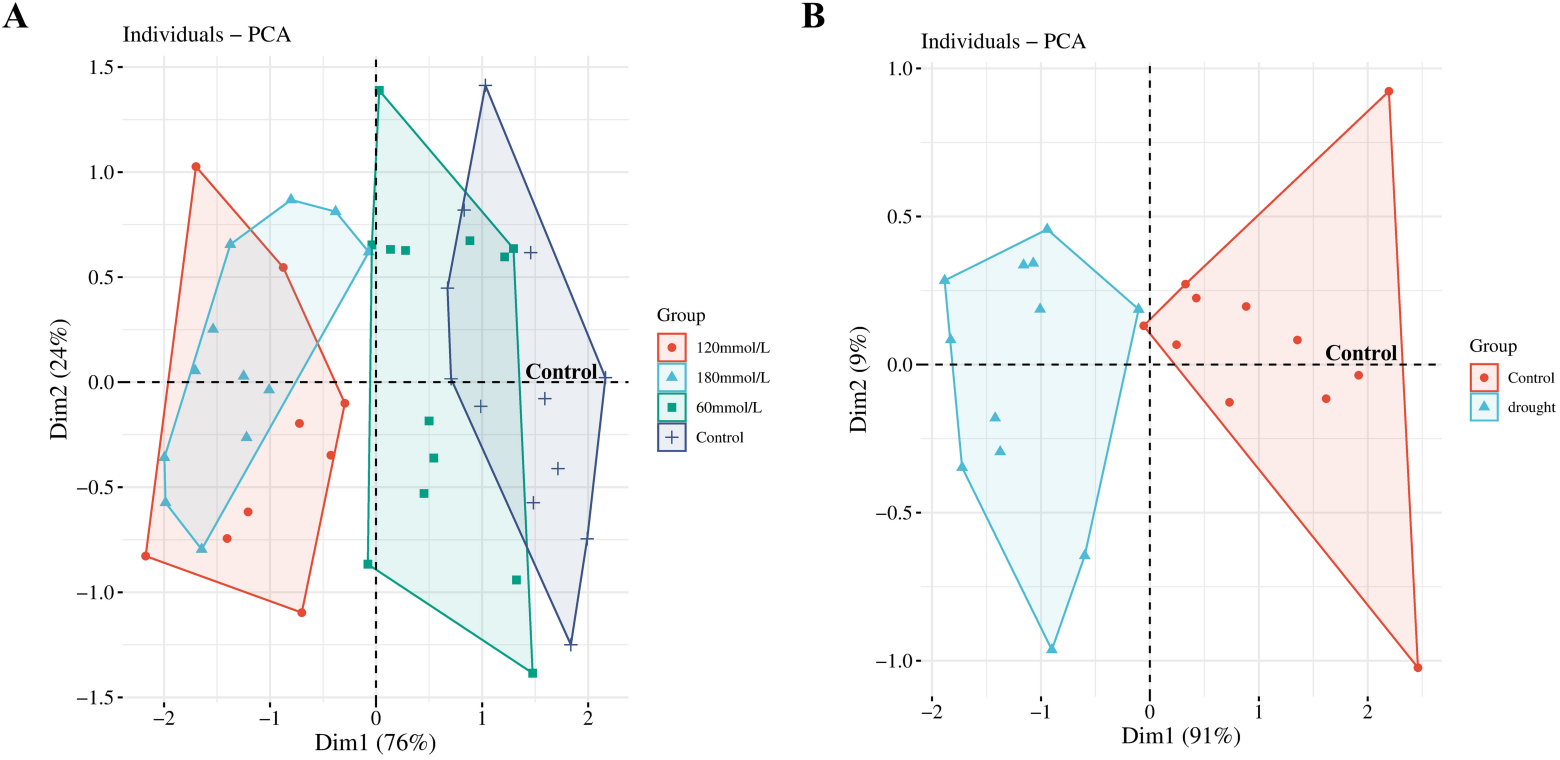
PCA scores obtained from the biophoton data. **(A)** PCA score plots of the biophoton characteristic obtained from samples at different salinities. **(B)** PCA score plots of the biophoton characteristic obtained from drought stress group.

PCA was then applied to provide a focused view of the variance in the biophoton characteristic parameters (I_0_ and CPS) to differentiate the stress subtypes. The results indicated that the biophoton characteristics could reasonably stratify the samples between the control group and the high salinity and drought stress groups (**Figure 6**).

**Figure 7:**
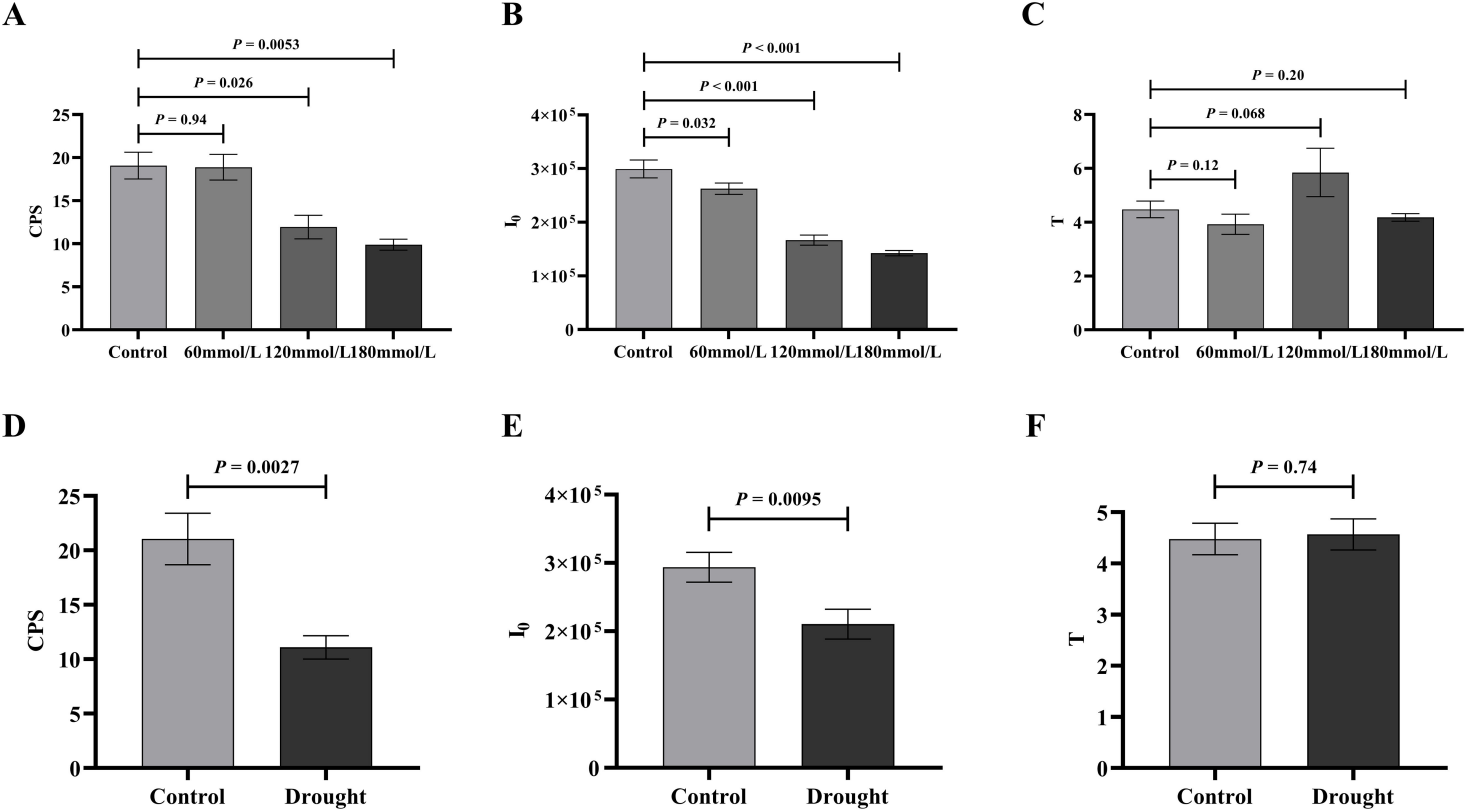
The t-test analysis of the biophoton parameters under different stress conditions. **(A–C)** The t-test analysis results of I_0_, CPS, and T among different salt concentration. **(D–F)** The t-test analysis results of I_0_, CPS, and T in different drought stress treatment group.

A two-tailed unpaired Student’s t-test was used to compare the differences in biophoton parameters between the control group and the stress treatment groups. The analysis results indicated significant differences in both I_0_ and CPS between the control group and the high salinity groups. The control group showed higher I_0_ and CPS than the high salinity groups, as shown in **Figures 7A, B**. Similar results were observed under drought stress groups, where I_0_ and CPS were significantly lower in the drought stress group compared to the control group (**Figures 7D, E**). However, there were no significant differences in T between any of these groups (**Figures 7C, F**).

### 3.3 The active ingredient content of *Isatidis Folium*

**Figure 8:**
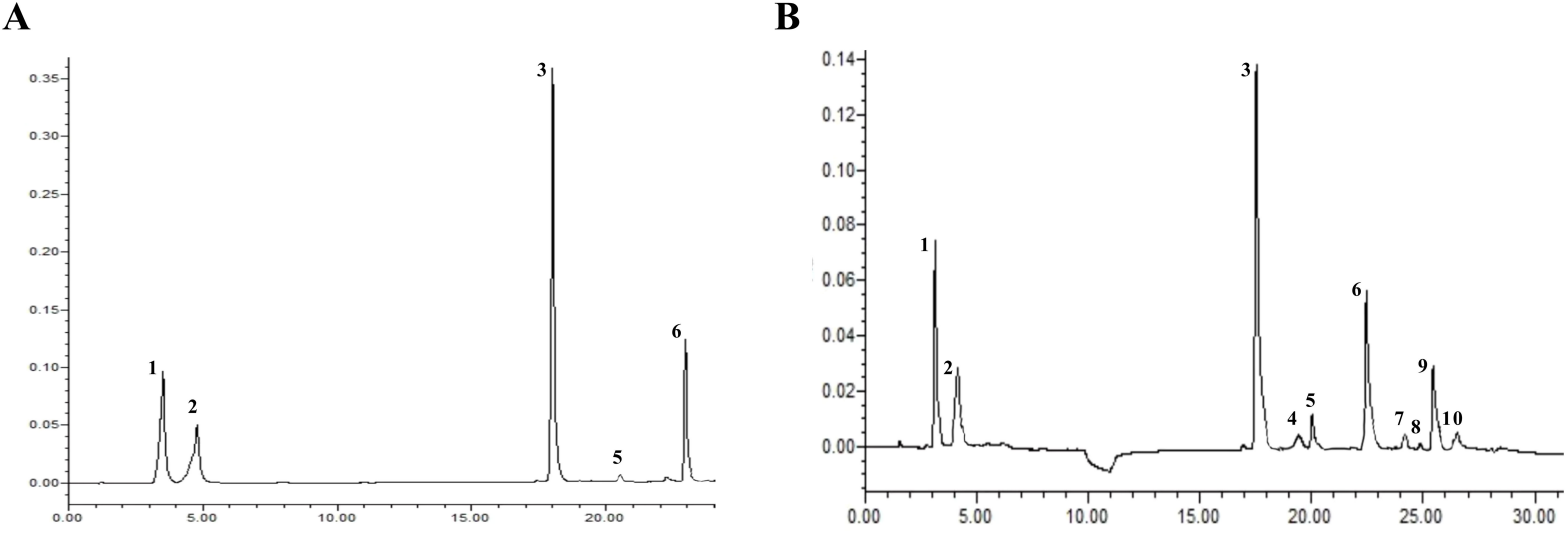
The UPLC chromatograms of *Isatidis Folium*. **(A)** The standard chromatogram. **(B)** The sample chromatogram. 1: 4-hydroxyquinazoline; 2: syringic acid; 3: tryptanthrin; 5: indigo; 6: indirubin. However, compounds 4, 7, 8, 9, and 10 were uncertain.

The active ingredient content in *Isatidis Folium* was determined by UPLC. Five peaks were identified by comparing the retention times of the samples (**Figure 8B**) with those of the standards (**Figure 8A**), including 4-hydroxyquinazoline, syringic acid, tryptanthrin, indigo, and indirubin. The precision ranged from 0.99% to 2.59% and the RSD of the reproducibility test ranged from 0.976% to 3.246%. The stability test was performed by analyzing the sample solution at six different time points after the preparation of the test sample (0, 4, 8, 12, 16, 20, and 24 hours) at room temperature, with RSDs ranging from 1.395% to 2.085%. Standard solutions at 0.5, 1, and 1.5 times the sample concentration were added to the samples, and the recovery test was then conducted according to the method described in Section “2.3.2”. The average recoveries ranged from 99.29% to 100.66% with RSDs from 1.72% to 4.32%. These results indicate the good performance and data reliability of the UPLC method.

**Figure 9:**
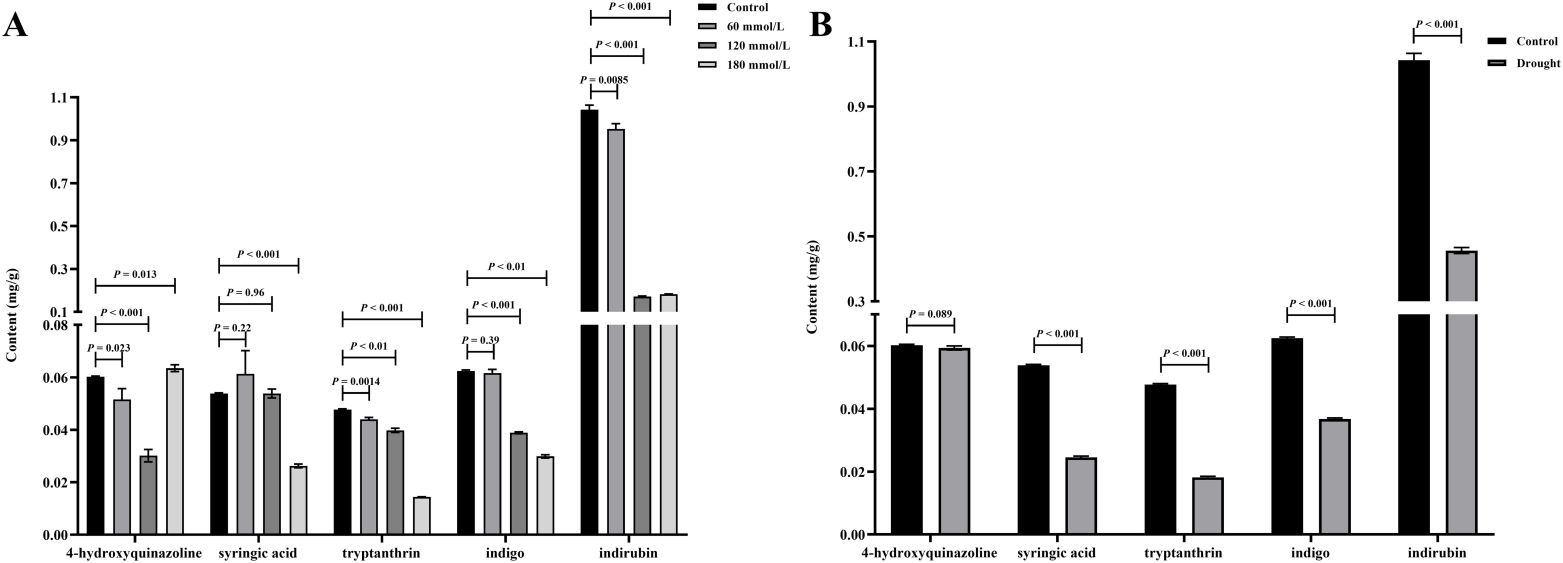
The t-test analysis results of the active ingredient content in *Isatidis Folium* under different stress conditions. **(A)** Salt stress; **(B)** Drought stress.

**Figure 9A** illustrates the differences in the levels of the five active ingredients under different salinity conditions. The t-test analysis results showed that except for the content of 4-hydroxyquinazoline, the levels of both tryptanthrin, syringic acid, indigo, and indirubin in the high salinity stress groups were significantly lower than those in the control group. Although the levels of certain constituents in the low salinity group also differed to some extent from those in the control group, the variability was small and the difference was not significant compared with the control group. **Figure 9B** illustrates the differences in the levels of the five active ingredients under different drought stress conditions. The t-test analysis results showed that the levels of syringic acid, tryptanthrin, indigo, and indirubin were all significantly lower in the drought stress group than in the control group. According to the 2020 edition of the Chinese Pharmacopoeia, the standard content of indirubin was 0.02%. The indirubin content was 0.1% in the control group, 0.046% in the drought group, 0.095% in the low salinity stress group, and about 0.018% in the high salinity stress group. Based on the levels of these five active ingredients, it can be concluded that the quality of *Isatidis Folium* varies under different environmental conditions. This suggests that *Isatis indigotica* Fort possesses a certain tolerance to salt and drought, while the accumulation of most of the active ingredients was strongly inhibited under severe salt stress.

### 3.4 Inhibitory activity of *Isatidis Folium* decoction against *E. coli* and *S. aureus*

**Table 1** shows the inhibitory activity of the *Isatidis Folium* decoction against *E. coli* and *S. aureus* under different stress conditions. In the control group, the decoction of *Isatidis Folium* was able to significantly inhibit the growth of *E. coli* and *S. aureus* at a concentration of 128-256 μg/mL, whereas in the high salinity and drought stress groups, a concentration of 512 μg/mL was required, and especially under drought stress, a concentration of 1024 μg/mL was required to inhibit the growth of *S. aureus*. The MIC values of both the high salinity group and the drought group were all significantly higher than those of the control group, indicating that the antibacterial activity of *Isatidis Folium* was significantly reduced under more severe stresses, implying that the medicinal effects were also severely inhibited.

**Table 1:** Inhibitory activity of *Isatidis Folium* decoction against *E. coli* and *S. aureus*.

Bacterial Species	Number	Stress Conditions	MIC (μg/mL)	Bacterial Species	Number	Stress Conditions	MIC (μg/mL)
*E coli.*	1	Control1	128	*S. aureus*	13	Control1	256
	2	Control2	256		14	Control2	256
	3	Control3	128		15	Control3	128
	4	Control4	128		16	Control4	128
	5	Drought1	512		17	Drought1	1024
	6	Drought2	512		18	Drought2	1024
	7	Drought3	512		19	Drought3	1024
	8	Drought4	512		20	Drought4	1024
	9	Salt1	512		21	Salt1	512
	10	Salt2	512		22	Salt2	512
	11	Salt3	512		23	Salt3	512
	12	Salt4	512		24	Salt4	512

### 3.5 Effects of different stress conditions on photosynthetic pigment content

**Figure 10:**
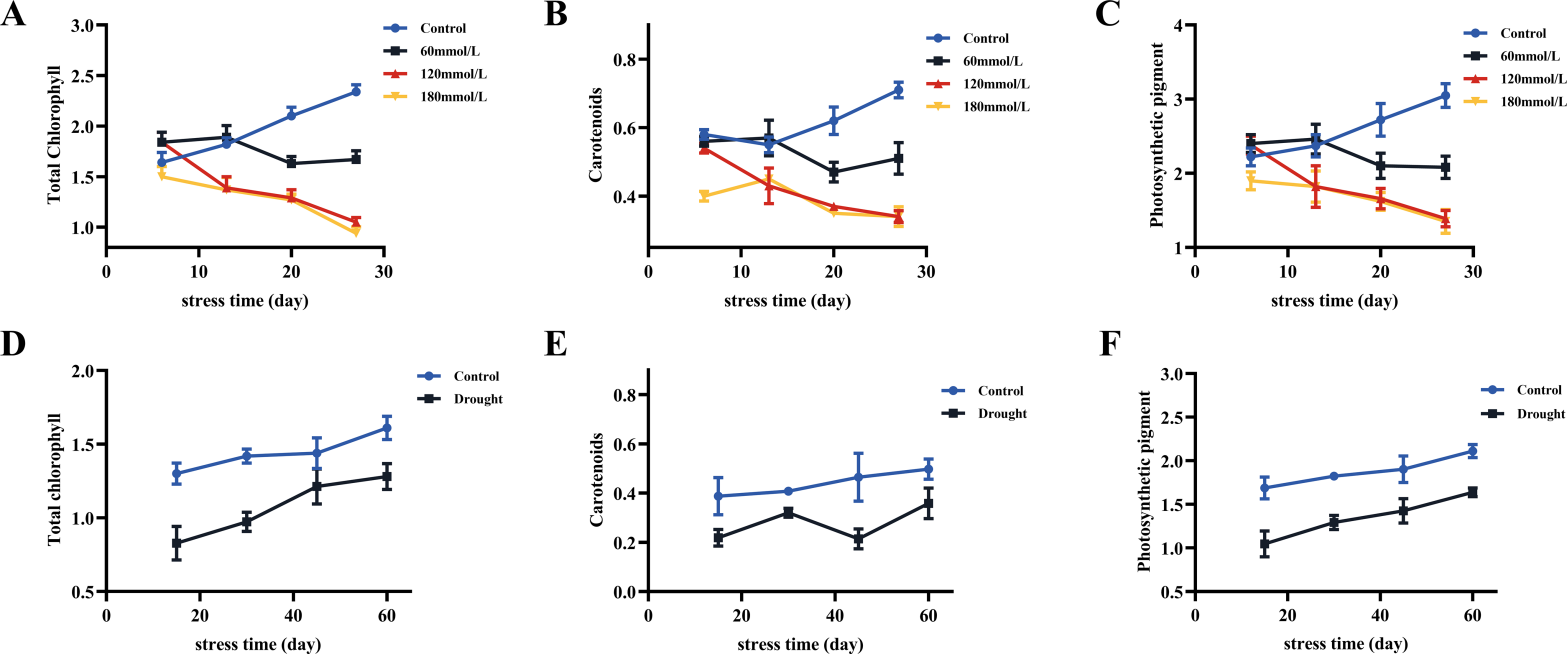
The content of total chlorophyll, carotenoids, and photosynthetic pigments in fresh *Isatis indigotica* Fort leaves under different stress conditions. **(A–C)** Salt stress. **(D–F)** Drought stress.

**Figure 11:**
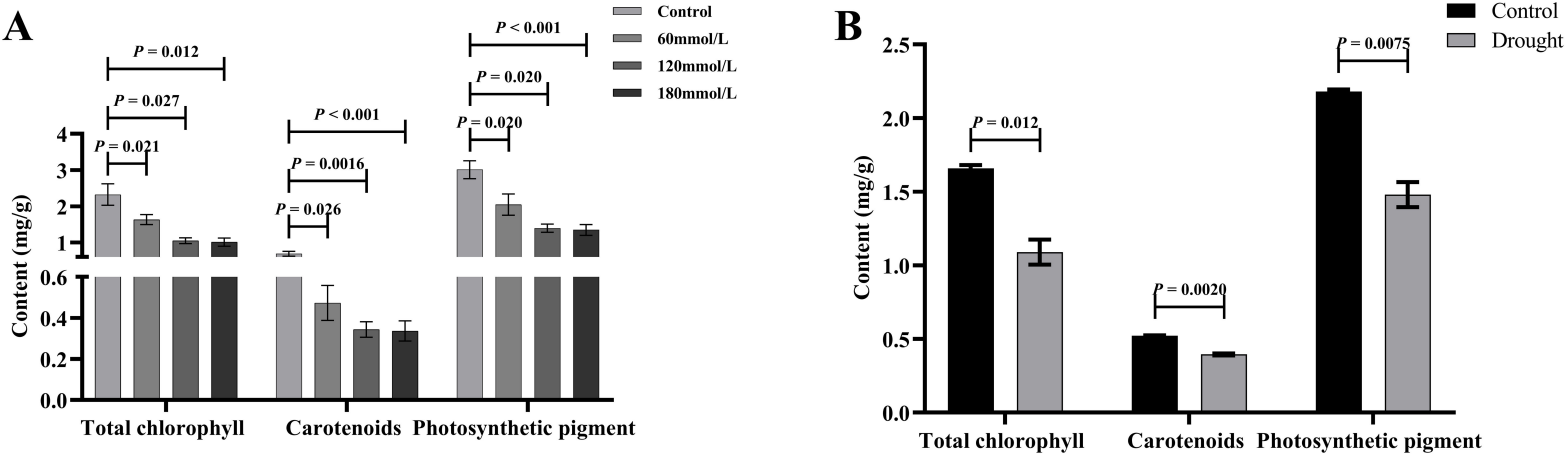
The t-test analysis results of total chlorophyll, carotenoids, and photosynthetic pigments content under different stress conditions. **(A)** Salt stress. **(B)** Drought stress.

As the salt stress progressed, the total chlorophyll and carotenoid contents of the control group all showed increasing trends, whereas the high salinity groups showed clear decreasing trends. At the same time, those of the high salinity groups were all lower than those of the control and the low salinity groups (**Figures 10A–C**). The contents of photosynthetic pigments in the low salinity group showed no difference compared to the control group in the early stage of stress, but the continuous accumulation of salt led to a decreasing trend in the later stage of stress. Based on the t-test analysis, in the later stage of stress, the contents of photosynthetic pigments of the control group were all significantly higher than those of the high salinity groups, as shown in **Figure 11A**. In addition, at the later stage of stress, the contents of photosynthetic pigments of the low salinity group were also lower than those of the control group, but the decreasing trend was relatively insignificant compared to those of the high salt stress groups. Under drought stress, the contents of photosynthetic pigments in both control and drought groups showed continuously increasing trends, but those of the control group were always significantly higher than those of the drought stress group (**Figures 10D–F**). **Figure 11B** shows the t-test analysis results that the contents of photosynthetic pigments were significantly different between the drought group and the control group, with the control group having significantly higher levels.

### 3.6 Effects of different stress conditions on REC

**Figure 12:**
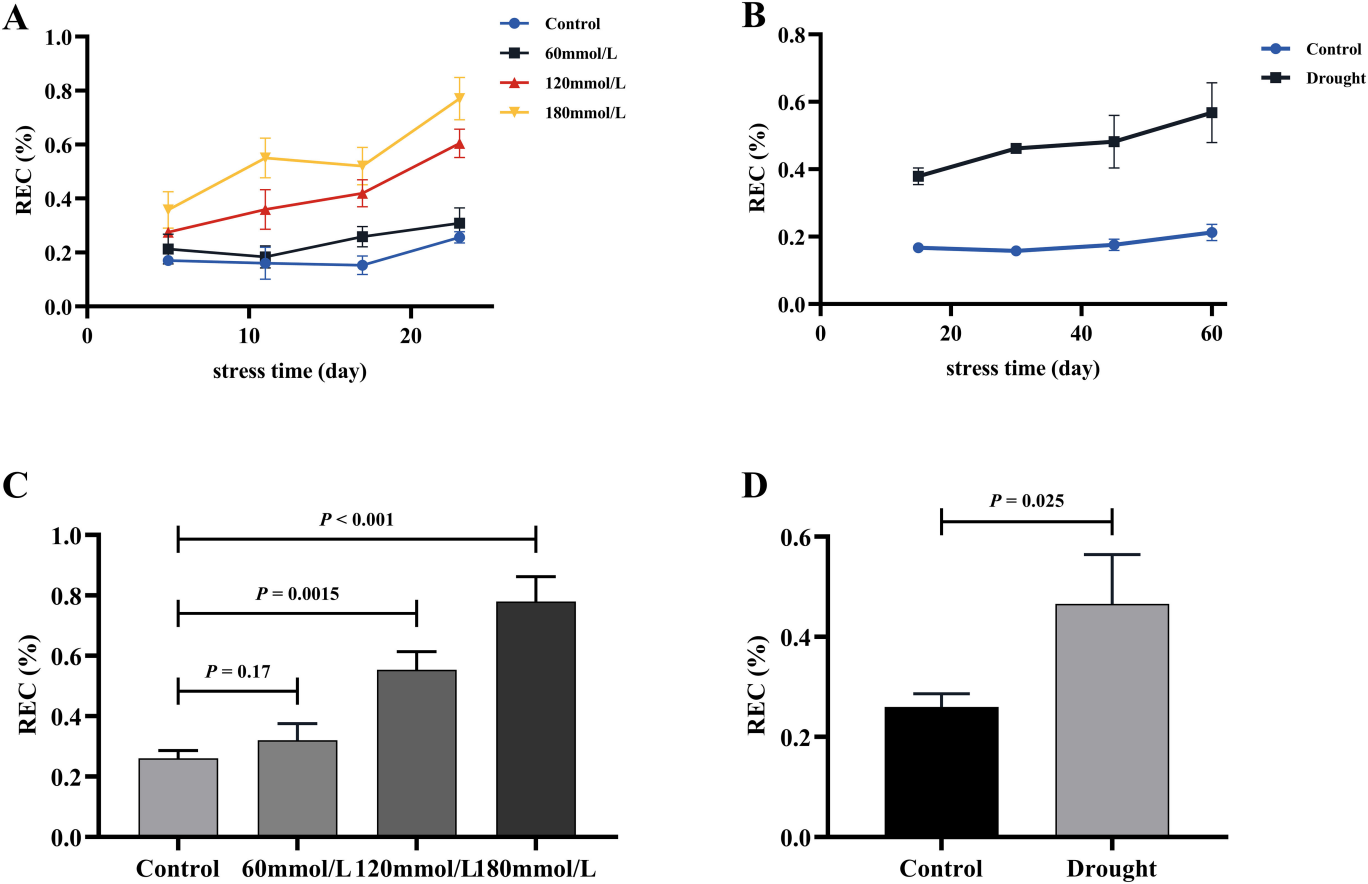
The REC of fresh *Isatis indigotica* Fort leaves under the two different treatment factors. **(A, B)** The variation of REC treated with salt stress and drought stress with the treatment time. **(C, D)** The t-test analysis results of REC in salt and drought stress groups.

**Figures 12A, B** illustrate the variations in REC under different stress conditions. As the stress progressed, the REC of the stress groups exhibited different degrees of increase, whereas the REC of the control group remained consistently lower than those observed in the stressed groups and were stable over time. **Figures 12C, D** show the results of the t-test analysis, indicating that the REC of the control group was significantly lower than that of the high salinity and drought groups. Specifically, the REC of the high salinity and drought groups were approximately 136.33%, 200.78%, and 167.67% higher than that of the control group.

### 3.7 Effects of different stress conditions on ROS production rate

**Figure 13:**
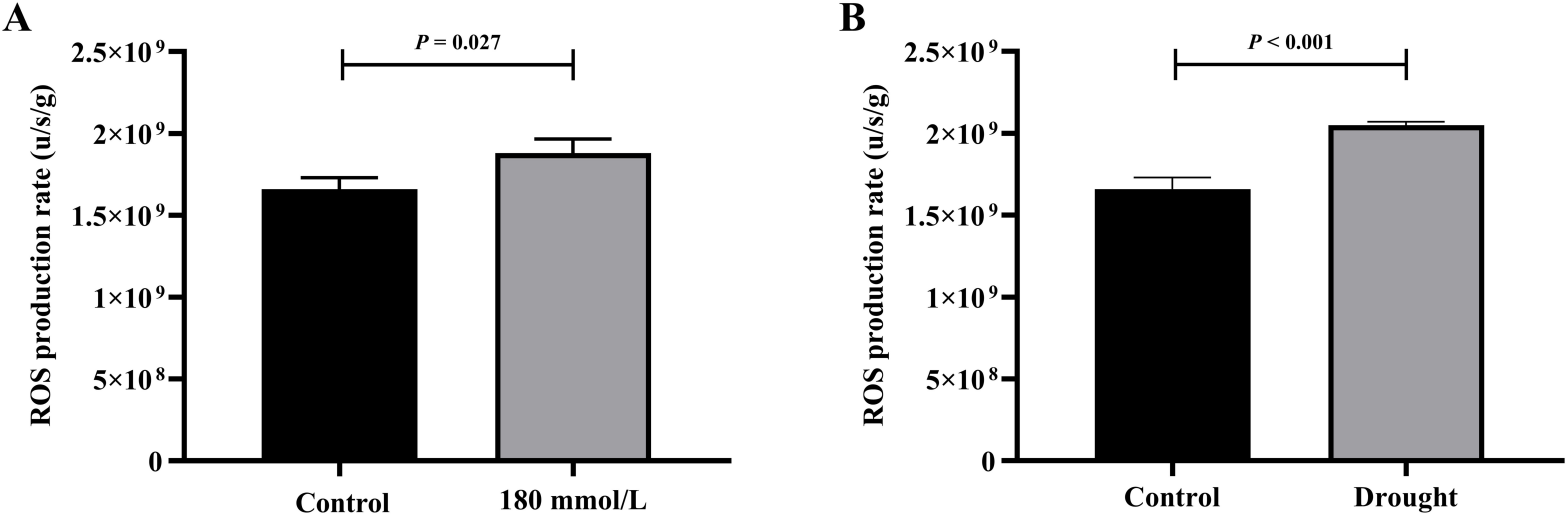
The t-test analysis results of ROS production rate under different stress conditions. **(A)** Salt stress. **(B)** Drought stress.

Among the salt stress groups, the control and 180 mmol/L groups differed significantly and were selected for the t-test analysis. **Figure 13** illustrates the differences in the production rate of ROS in the different stress groups: the rates in both the 180 mmol/L group and the drought group were all significantly higher than those in the control group.

### 3.8 Correlation analysis

**Figure 14:**
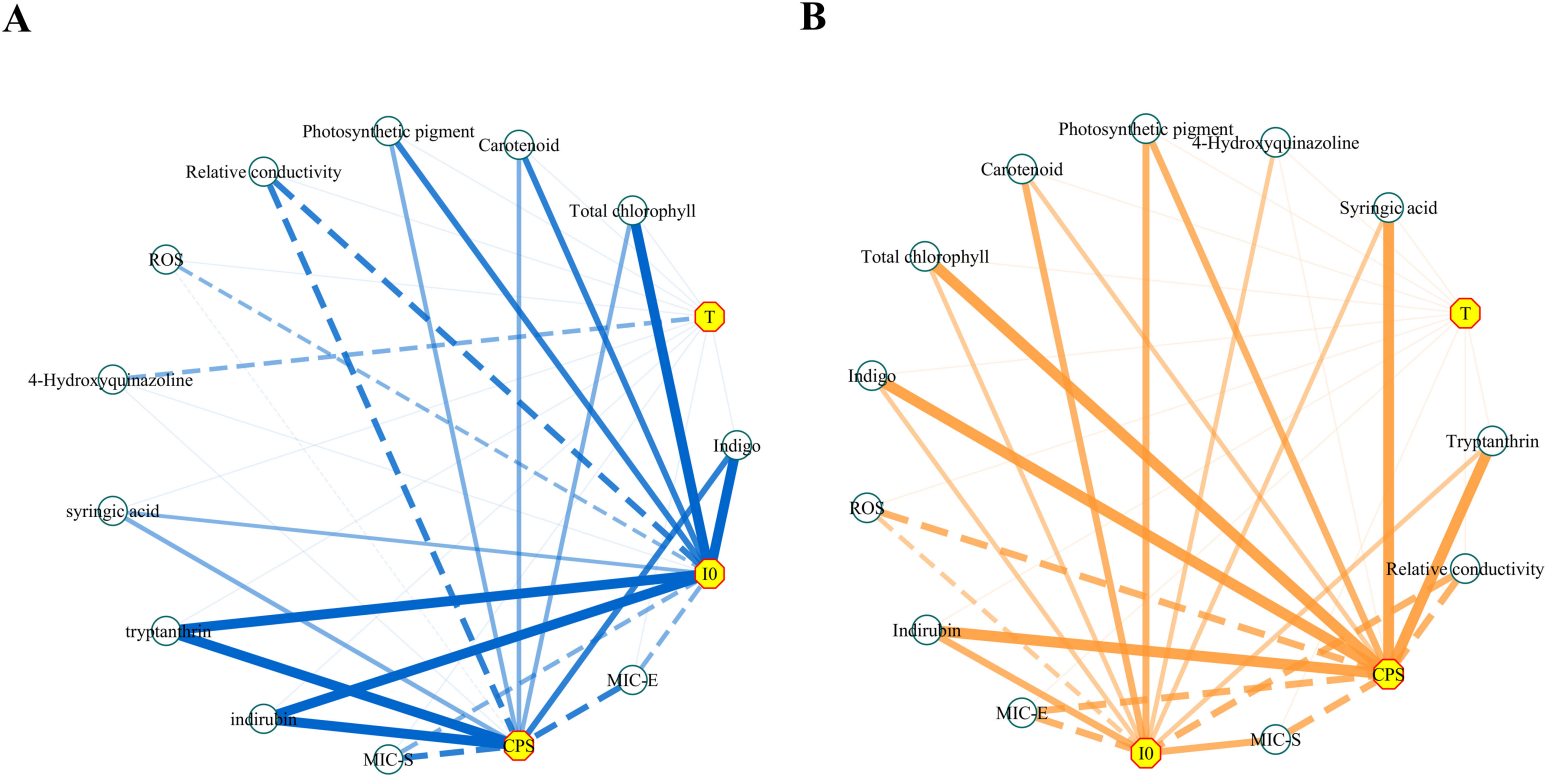
The network diagrams of the correlation analysis. **(A)** Salt stress. **(B)** Drought stress. Negative correlations were indicated by dotted lines and positive correlations were indicated by solid lines; the thicker lines indicated a higher correlation (i.e., a larger Spearman’s |ρ|). The length of each line had no meaning. MIC-E and MIC-S represent the minimum concentration at which the decoction of *Isatidis Folium* significantly inhibited the growth of *E*. *coli* and *S. aureus*, respectively.

Based on the preceding results, the correlation between biophoton parameters and other indices was analyzed. The correlation analysis results are presented in **Table 2** and **Figure 14** provides a more intuitive view of the correlation network. The results indicated a positive correlation between the biophoton parameters (I_0_ and CPS) and the content of photosynthetic pigments and active ingredients such as syringic acid, tryptanthrin, indigo, and indirubin under different stress treatments. In addition, I_0_ and CPS were negatively correlated with REC, ROS generation rate, and MIC values. However, the correlation between T and other indicators was basically not significant.

**Table 2:** Correlation analysis results.

Biophoton parameter		CPS		I0		T	
Pearson *r*/*P* value		*r*	*P*	*r*	*P*	*r*	*P*
Salt stress	4-Hydroxyquinazoline	0.263	0.4088	0.429	0.1641	-0.7817	0.0027**
	Syringic acid	0.7413	0.0058**	0.6071	0.0363*	0.09592	0.7668
	Tryptanthrin	0.9091	<0.0001****	0.9487	<0.0001****	-0.2543	0.4251
	Indigo	0.8807	0.0002***	0.9545	<0.0001****	-0.5473	0.0655
	Indirubin	0.8982	<0.0001****	0.9735	<0.0001****	-0.4665	0.1263
	Total chlorophyll	0.8092	0.0026**	0.9132	<0.0001****	-0.3809	0.2478
	Carotenoid	0.7555	0.0072**	0.8814	0.0003***	-0.336	0.3123
	Photosynthetic pigment	0.7991	0.0032**	0.9082	0.0001***	-0.3717	0.2604
	Relative conductivity	-0.8514	0.0004***	-0.8808	0.0002***	0.186	0.5628
	ROS production rate	-0.7326	0.0977	-0.9007	0.0143*	-0.4926	0.3209
	MIC-E	-0.9280	<0.0001***	-0.9564	<0.0001**	0.3617	0.2480
	MIC-S	-0.9600	<0.0001***	-0.9224	<0.0001**	0.2810	0.3763
Drought stress	4-Hydroxyquinazoline	0.6215	0.1878	0.8299	0.0409*	-0.3934	0.4403
	Syringic acid	0.9811	0.0005***	0.909	0.0121*	-0.1723	0.7441
	Tryptanthrin	0.9812	0.0005***	0.916	0.0103*	-0.1793	0.7339
	Indigo	0.9825	0.0005***	0.9163	0.0102*	-0.1778	0.7361
	Indirubin	0.9818	0.0005***	0.9258	0.0081**	-0.2025	0.7003
	Total chlorophyll	0.9757	0.0009***	0.9119	0.0113*	-0.2219	0.6727
	Carotenoid	0.8359	0.0382*	0.97	0.0013**	-0.5034	0.3086
	Photosynthetic pigment	0.967	0.0016**	0.9528	0.0033**	-0.3014	0.5616
	Relative conductivity	-0.9319	0.0068**	-0.9436	0.0047**	0.4321	0.3922
	ROS production rate	-0.9414	0.005**	-0.9141	0.0107*	0.2189	0.6768
	MIC-E	-0.8682	0.0003**	-0.7302	0.0070**	0.1737	0.5893
	MIC-S	-0.9490	<0.0001**	-0.8916	<0.0001**	0.4021	0.1951

MIC-E and MIC-S represent the MIC values of *Isatidis Folium* decoction against *E coli.* and *S. aureus*, respectively. *P < 0.05, **P < 0.01, ***P < 0.001, and ****P < 0.0001.

## 4 Discussion

According to the metabolic luminescence theory, redox reactions in biological systems produce highly reactive peroxyl radicals, which can then be oxidized into excited-state biomolecules. These inherently unstable biomolecules must return to the ground state, releasing excess energy in the form of photons during these transitions (Cifra and Pospísil, 2014). It is reasonable to assume that a biological system with higher metabolic activity will contain more biomolecules in the excited state. The greater the energy released by these active biomolecules during energy level transitions, the greater the intensity of the biophoton emission. Plant stress exposure leads to ROS accumulation, disrupting cell membrane structure and function, and promoting the formation of harmful compounds like superoxide anion and MDA. Such disruptions can lead to metabolic disruption and, ultimately, a decrease in the intensity of biophoton emission (Bu et al., 2022). It was found that the initial delayed fluorescence of winter wheat decreased by an order of magnitude under stress conditions (Jócsák et al., 2024). Consistent findings were obtained in our study: under high salinity and drought stress, the I_0_ of *Isatis indigotica* Fort leaves was approximately halved relative to the control group. Additionally, evidence indicates that I_0_ levels can reflect the metabolic strength of biological systems (Xi et al., 2011; Gang et al., 2015), which supports our previous view regarding the correlation between metabolic strength and biophoton emission intensity. Thus, it can be postulated that high salt and drought stresses likely caused significant cellular damage, inhibiting metabolic activity in *Isatis indigotica Fort* leaves. In the study of the DL characteristics, in addition to the intensity parameter, the time parameter T, introduced by Professor Gu, serves as an important macroscopic indicator reflecting the decay rate of the DL kinetic curve. Mathematical analysis of the DL decay kinetics revealed no significant differences in T between different stress conditions. This implies that different stress conditions had minimal impact on the decay characteristics of the DL kinetic curves of samples. Similar results were observed in a related study (Gu, 2012), where Europe *Aloe vera* samples showed a 30% higher I_0_ than South America samples, yet their T values remained nearly identical. This indicates that *Aloe vera* samples from these two regions differed in the “density” category, but were similar in the “composition” category. Consequently, we infer that *Isatis indigotica* Fort leaves subjected to different stress conditions maintained a similar level of “composition”, resulting in stable T values. However, stress influenced the “density”, such as the content of constituent substances, leading to a significant difference in I_0_ values. Therefore, we believe that, although parameter T cannot be used as a sensitive indicator to distinguish *Isatis indigotica* Fort leaves under various stress conditions, it can still reflect this valuable information.

These two severe abiotic stresses can promote the excessive accumulation of ROS and lead to oxidative stress damage. Such perturbations can lead to the extravasation of intracellular substances and the degradation of chlorophyll, thereby affecting physiological and metabolic processes such as photosynthesis (Sun et al., 2012). Chloroplasts are the sites of photosynthesis and can use photosynthetic pigments to convert light energy into chemical energy and store it in organic matter, which is the energy source for all life activities in green plants. The structure of the chloroplast remains relatively stable under normal physiological conditions (Qu et al., 2024). However, under stress conditions, the normal structure of the chloroplast is disrupted, leading to a series of abnormal phenomena, including plasmalemma separation, disorganization of internal components, and rupture of the membrane system (Long and Deng, 2019). This leads to the degradation of chlorophyll, disruption of physiological functions, and inhibition of photosynthesis. Consequently, the destruction of chloroplast structure and function will inevitably weaken the metabolic activities of green plants and inhibit their normal growth. Therefore, in this study, we developed these two stress models to investigate the potential relationship between biophoton emission characteristics, physiological state, and herbal quality. The test results showed that the photosynthetic pigment content of *Isatis indigotica* Fort leaves decreased significantly under stress conditions. This suggests that salt and drought stress could damage the chloroplasts, resulting in reduced energy production and unfavorable secondary metabolite synthesis. The study also showed that the REC of samples under both high salinity stress and drought stress were all significantly higher than that of the control group. This suggests that under stress conditions, the cell membrane structure suffers severe damage, resulting in a loss of the ability to regulate the entry and exit of substances. At the same time, the stress disrupts the dynamic balance between ROS generation and consumption in the leaves, leading to membrane rupture and metabolic disorders (Meng et al., 2022). As a result, plant growth is inhibited, inevitably affecting the accumulation of active ingredients.

The correlation analysis revealed a strong correlation between the physiological indicators and the biophoton parameters (I_0_ and CPS). Compared to the control group, there was a significant decrease tread in photosynthetic pigment content with increasing stress levels. I_0_ and CPS positively correlated with photosynthetic pigment content, consistent with prior research findings (Sun et al., 2023b). Lower chlorophyll content correlates with reduced photosynthesis, indicating weakened metabolic activity and decreased biophoton emission intensity. In contrast, both I_0_ and CPS showed a significant negative correlation with the rate of ROS production, which seems to contradict some reports in the literature. This discrepancy may be due to the source of biophoton emission in *Isatis indigotica* Fort leaves differing from that observed in other sample types. It is well known that ROS is one of the major sources of ultra-weak luminescence and that the ultra-weak luminescence of biological samples is positively correlated with oxidative stress. However, ROS may not be the only or direct source of the changes in biophoton emission. On the contrary, some studies (Sun et al., 2023b; Bai et al., 2018b) have reported that the ultra-weak luminescence of green plants was negatively correlated with ROS, which is consistent with our results. This is because salt stress severely inhibits the photosystem (PS) activity and the oxygen-evolving complex in leaves, damages the integrity of the thylakoid membrane, decreases the photochemical efficiency of PSII, and hinders the QA-QB electron transfer (Sun et al., 2023a). This change inhibits photosynthesis and reduces the metabolic strength of the system. At the same time, the large accumulation of ROS under stress will damage the cell membrane system, causing extravasation of intracellular tissue and disorganization of tissue structure, leading to metabolic disorders, reduced growth and vigor, and ultimately reflected in the weakening of the CPS (Bai et al., 2018a). REC serves as a crucial physiological indicator of plant resistance, exhibiting a negative correlation with the structural integrity of cell membranes. The significant negative correlation between biophoton parameters and REC indicates that biophoton emission could reflect the extent of cell membrane damage in *Isatis indigotica* Fort leaves under stress. In conclusion, the results demonstrate that biophoton parameters can be used as effective indicators for reflecting the physiological status of *Isatis indigotica* Fort leaves.

Chinese herbs are composed of a variety of complex chemical components. Some of these components, such as alkaloids, flavonoids, glycosides, volatile oils, amino acids, possess specific biological activities and are collectively termed the active ingredients of Chinese herbs. Relevant studies have shown that, in addition to growth, appearance, and physiological indices, stressors can also affect the accumulation of active ingredients in medicinal plants (Tang et al., 2018; Yang et al., 2023). For medicinal plants, the content of active ingredients plays a crucial role in determining their quality. Consequently, excessive stress will inevitably affect the quality of the herbs. The results of the UPLC-based determination of active ingredients indicated that the effect of low salinity stress on the total active ingredient content of *Isatidis Folium* was not discernible. In contrast, under high salinity and drought stress conditions, a significant reduction in the content of the four main active ingredients was observed, which is consistent with the results reported in the literature (Zhou et al., 2019; Cheng, 2018). Similar results were also obtained in the determination of antibacterial activity: the MIC values of *E. coli* and *S. aureus* were significantly higher than those of the control group in both high salinity and drought stress groups, indicating that excessive stress also inhibited the antibacterial activity of *Isatidis Folium*. Combining the results of UPLC and MIC determination, we tentatively conclude that stress conditions, especially excessive abiotic stresses, can seriously reduce the herbal quality. Therefore, there is a significant difference between the herbal quality of our established stress models and the control group.

It is known that the form of excitation energy generated by light irradiation depends on the absorption of this energy by molecules within the body. Changes in the chemical composition and content of the herbs will alter the internal structure of the herbs, which in turn will alter the ability of the molecules to interact, absorb, and store excitation energy, resulting in different DL kinetics. In addition, some studies (Xi et al., 2015) have shown that SPE mainly occurs during the redox metabolism and protein synthesis processes in Chinese herbs, and the differences in the active ingredient content will affect the protein synthesis mechanism and related energy metabolism in the plants, thereby affecting the CPS of the Chinese herbs. The findings indicated that the content of the active components of *Isatidis Folium* under both two types of stress was positively correlated with the biophoton parameters (I_0_ and CPS). The levels of the active ingredients of *Isatidis Folium* under high salt and drought stress decreased significantly compared to the control group. It is important to note that *Isatidis Folium* contains mainly indigo, indirubin, tryptanthrin, and syringic acid, as active compounds, which may directly influence the pharmacological action and thus affect its therapeutic properties.

The DL technique has previously been identified as an effective method for quality control of Chinese herbal medicines, based on studies analyzing the DL characteristics, active ingredient content, and pharmacological activity. These studies have demonstrated an obvious correlation between biophoton parameters and quality indicators (Jia et al., 2020; Sun et al., 2019b, a, Sun et al., 2016b, a, Sun et al., 2020). Existing literature further indicates potential correlations between the biophoton emission characteristics and the herbal content of the active ingredients in fresh samples of several Chinese herbal plants, such as the roots of *Salvia miltiorrhiza* Bunge and *Platycodon grandiflorus* (Jacq.) A.DC (Zhao et al., 2017), *Lonicera japonica* Thunb. buds (Zhao, 2014), and *Leonurus japonicus* Houtt. leaves and *Carthamus tinctorius* L. flowers (Cao et al., 2023). All these studies provide a solid foundation for our exploration. Our study reveals that the biophoton characteristics of fresh *Isatis indigotica* Fort leaves of different stress conditions were significantly different and correlated with the active ingredient content, antibacterial activity, and physiological indices. Therefore, we preliminary believe it is feasible to use the biophoton characteristics of fresh *Isatis indigotica* Fort leaves to predict and evaluate the quality of *Isatidis Folium* during cultivation. Specifically, the biophoton detection technology enables real-time monitoring of *Isatidis Folium* quality during the growth of *Isatis indigotica* Fort. This allows for the timely removal of poor-quality plants or the implementation of corrective measures, thereby improving overall yield and quality.

In addition, other existing quality control methods of Chinese herbs, although mainstream, lack complete and accurate characterization. These methods rely on the analysis of dried Chinese herbs after processing, involving complex procedures that result in irreversible damage and waste. Additionally, they struggle to effectively monitor herbal quality during cultivation. Conversely, biophoton emission captures comprehensive intrinsic information from biological samples, aligning with the holistic view of traditional Chinese medicine and offering a more thorough reflection of herbal quality. Moreover, quality evaluation of Chinese herbs based on biophoton technology will not damage the structure or efficacy of the samples. The measurement of specific parameters by this technique allows quality to be determined immediately post-harvest. Therefore, biophoton detection technology has the advantages of rapidity, sensitivity, and non-invasiveness, simplifying the evaluation process compared to the traditional methods and requiring lower operational techniques and testing costs. However, given the limited sample diversity and size, further extensive experimental investigation is required to validate the generalizability and applicability of these findings. Due to the large equipment, many components, and complicated wiring, the current biophoton detectors can only be fixed in one place, making it impossible to detect fresh samples directly in the planting fields without removing the samples from the plants. The diversity of DL kinetic curve fitting methods results in a wide range of biophoton parameters and the lack of agreement in this domestic and international research field, which also leads to uncertainty and complexity in parameter selection. Spectroscopic detection techniques are also of great value in the characterization and identification of Chinese herbs (Yao et al., 2022; Qi et al., 2022), and when combined with modern scientific techniques such as chromatography and mass spectrometry, they can evaluate the properties of Chinese herbs more comprehensively. By combining HPLC with near-infrared technology and chemometric modeling, a comprehensive evaluation of Danshen medicines was achieved, from active ingredient quantification to overall quality variation (Li et al., 2022). The creation of a comprehensive analysis process of multispectral data enables the traceability of the origin of Chinese herbs (Qi et al., 2020). It not only helps to evaluate herbal quality but also facilitates similar quality assessment efforts for other natural medicines. Moreover, the spectroscopic method has the advantages of high sensitivity, high accuracy, and intelligence, which can achieve rapid, non-destructive, and environmentally friendly detection of Chinese herbs without affecting their quality and utility as well. Therefore, further identification of the most applicable fitting methods and characteristic parameters, as well as the development of portable instrumentation that can detect the light intensity and spectrum concurrently, is crucial for the real promotion of this technology for quality prediction and monitoring during herbal cultivation. If this technology is to be truly promoted and applied to quality prediction and monitoring during the cultivation of Chinese herbs, future research should focus on establishing mathematical models of biophoton parameters based on multiple sources and large samples, to better guide practical applications.

## 5 Conclusion

The quality of Chinese herbs represents a pivotal concern in the modernization and internationalization of traditional Chinese medicine. Currently, the quality of *Isatidis Folium* varies, and the evaluation system requires improvement. In this study, we proposed a novel method to identify different stress subtypes of *Isatis indigotica* Fort using biophoton technology and preliminarily verified its feasibility in the quality evaluation of *Isatidis Folium*, which aligns with the holistic perspective of traditional Chinese medicine and is also simple and rapid. This study revealed significant differences in the biophoton parameters (I_0_ and CPS) and physiological indices of fresh *Isatis indigotica* Fort leaves and in the active ingredient content and antibacterial activity of *Isatidis Folium* under various stress conditions. I_0_ and CPS were able to accurately differentiate between stress subtypes and the growth status of *Isatis indigotica* Fort and were correlated with the quality of *Isatidis Folium*. Thus, we believe that the biophoton detection technique has good potential as a rapid technique to predict and evaluate the quality of *Isatidis Folium*. Furthermore, I_0_ and CPS may serve as effective indicator parameters with significant potential for herbal quality monitoring.

## Data Availability

The raw data supporting the conclusions of this article will be made available by the authors, without undue reservation.
